# Isolation and characterization of the *E. coli* membrane protein production strain Mutant56(DE3)

**DOI:** 10.1038/srep45089

**Published:** 2017-03-24

**Authors:** Thomas Baumgarten, Susan Schlegel, Samuel Wagner, Mirjam Löw, Jonas Eriksson, Ida Bonde, Markus J. Herrgård, Hermann J. Heipieper, Morten H. H. Nørholm, Dirk Jan Slotboom, Jan-Willem de Gier

**Affiliations:** 1 Center for Biomembrane Research, Department of Biochemistry and Biophysics, Stockholm University, Stockholm, SE-106 91, Sweden; 2 Technical University of Denmark, Novo Nordisk Foundation Center for Biosustainability, Kogle Alle 6, Hørsholm, 2970, Denmark; 3 Helmholtz Centre for Environmental Research-UFZ, Department of Environmental Biotechnology, Permoserstrasse 15, Leipzig, 04318, Germany; 4 Groningen Biomolecular Science and Biotechnology Institute, University of Groningen, Nijenborgh 4, Groningen, AG, 9747, The Netherlands

## Abstract

Membrane protein production is usually toxic to *E. coli*. However, using genetic screens strains can be isolated in which the toxicity of membrane protein production is reduced, thereby improving production yields. Best known examples are the C41(DE3) and C43(DE3) strains, which are both derived from the T7 RNA polymerase (P)-based BL21(DE3) protein production strain. In C41(DE3) and C43(DE3) mutations lowering *t7rnap* expression levels result in strongly reduced T7 RNAP accumulation levels. As a consequence membrane protein production stress is alleviated in the C41(DE3) and C43(DE3) strains, thereby increasing membrane protein yields. Here, we isolated Mutant56(DE3) from BL21(DE3) using a genetic screen designed to isolate BL21(DE3)-derived strains with mutations alleviating membrane protein production stress other than the ones in C41(DE3) and C43(DE3). The defining mutation of Mutant56(DE3) changes one amino acid in its T7 RNAP, which weakens the binding of the T7 RNAP to the T7 promoter governing target gene expression rather than lowering T7 RNAP levels. For most membrane proteins tested yields in Mutant56(DE3) were considerably higher than in C41(DE3) and C43(DE3). Thus, the isolation of Mutant56(DE3) shows that the evolution of BL21(DE3) can be promoted towards further enhanced membrane protein production.

The natural abundance of membrane proteins is typically too low to isolate sufficient amounts of material for functional and structural studies^1^. Therefore, membrane proteins must be obtained by producing them recombinantly and the bacterium *Escherichia coli* has been widely used for this purpose^2^. Despite tremendous efforts, there are very few examples of membrane proteins that have been successfully refolded after denaturing isolation from inclusion bodies^1^. Therefore, it is preferred to produce membrane proteins in the cytoplasmic membrane of *E. coli* from which they can be purified after detergent extraction^1^. Unfortunately, the production of membrane proteins in the cytoplasmic membrane is generally toxic to *E. coli*^3^,^4^. However, using gentic screens strains can be isolated in which the toxicity of membrane protein production is reduced resulting in improved yields^5^,^6^,^7^,^8^,^9^. Best known examples are the C41(DE3) and C43(DE3) strains, which are both derived from the BL21(DE3) protein production strain^5^. Ever since the isolation of C41(DE3) and C43(DE3) some twenty years ago, no efforts have been reported to isolate BL21(DE3)-derived strains with further improved membrane protein production characteristics. Here, our aim was to explore if such strains can be isolated.

BL21(DE3) is the most widely used *E. coli* host for the production of proteins^10^. In BL21(DE3), expression of the gene encoding the target protein is driven by the chromosomally encoded bacteriophage T7 RNA polymerase (P), which transcribes eight times faster than *E. coli* RNAP^11^,^12^,^13^. T7 RNAP specifically recognizes the T7 promoter, which drives the expression of the target gene from a plasmid^11^,^13^. The gene encoding the T7 RNAP is under control of the *lac*UV5 promoter region (P_*lac*UV5_), which is a strong variant of the wild-type *lac* promoter (P_*lac*WT_)^14^,^15^. The Lac-repressor, LacI, binds to the operator site in P_*lac*UV5_^16^. Isopropyl-β-D-thiogalactopyranoside (IPTG) mediates the dissociation of LacI from the operator site^16^. Thus, expression of the gene encoding the T7 RNAP can be induced with IPTG, which leads to the synthesis of T7 RNAP and consequently target gene expression. To isolate C41(DE3), BL21(DE3) was transformed with a T7-based expression vector harboring the gene encoding the mitochondrial oxoglutarate malate carrier protein (OGCP), whose expression is extremely toxic to BL21(DE3)^5^. Expression of *ogcp* was induced in liquid culture with IPTG and surviving cells were selected for on IPTG-containing agar plates. Clones that were IPTG resistant on plate and efficiently produced OGCP upon the addition of IPTG were cured from the expression plasmid. This resulted in the isolation of C41(DE3). Recently, we have shown that the defining mutations in C41(DE3) weaken the promoter governing *t7rnap* expression^17^,^18^. In C41(DE3), P_*lac*UV5_ is partially mutated back to P_*lac*WT_ resulting in a promoter that is not only less strong than P_*lac*UV5_, but also less strong than P_*lac*WT_^18^. Therefore, the promoter governing *t7rnap* expression in C41(DE3) was denoted P_*lac*Weak_^18^. P_*lac*Weak_ governing the expression of *t7rnap* leads to less T7 RNAP synthesized and consequently lower target gene expression intensities^17^. Although maybe in first instance counterintuitive, lower target gene expression intensities lead for many membrane proteins to more efficient production in the cytoplasmic membrane because saturation of the membrane protein biogenesis machinery is prevented^17^,^19^. Recently, we have shown that the conversion of P_*lac*UV5_ to P_*lac*Weak_ in C41(DE3) occurred through RecA-dependent recombination of P_*lac*UV5_ with P_*lac*WT_ of the *lac*-operon^18^. Furthermore, it was shown that these mutations are selected for upon the production of almost any recombinant protein and that the mechanism driving these mutations provides BL21(DE3) with a very efficient way to alleviate protein production stress^18^. C43(DE3) was derived from C41(DE3) using a similar setup used for the isolation of C41(DE3) from BL21(DE3)^5^. For the isolation of C43(DE3), a target membrane protein was used that was even toxic to C41(DE3). It has been shown that C43(DE3) has accumulated mutations in the gene encoding LacI that is adjacent to *t7rnap*^20^. It has been proposed that the mutated LacI binds more tightly to the *lac*-operator, resulting in an even more tightly regulated expression of *t7rnap*^20^. Both C41(DE3) and C43(DE3) have been widely and very successfully used to produce a plethora of membrane proteins for functional and structural studies^21^. In our attempts to isolate BL21(DE3)-derivatives with even further enhanced membrane protein production characteristics than the ones of the C41(DE3) and C43(DE3) strains, we used a simple plate-based genetic screen. An exceptionally toxic target membrane protein that, in contrast to other targets, has so far not led to the isolation of mutations in P_*lac*UV5_ was used to screen for IPTG-resistant BL21(DE3)-derivatives^18^. This toxic target membrane protein was fused to green fluorescent protein (GFP) enabling the rapid identification of IPTG-resistant clones efficiently producing the target in the cytoplasmic membrane already on plate. This setup led to the isolation of a BL21(DE3)-derivative that produces many membrane proteins to higher levels than C41(DE3) and C43(DE3) as well as their ancestor BL21(DE3). This BL21(DE3)-derivative was denoted Mutant56(DE3), which is hereafter referred to as Mt56(DE3). After its isolation, we sent Mt56(DE3) to a few structural biology laboratories for unbiased testing^22^,^23^,^24^,^25^. Mt56(DE3) performed really well; it was *e.g*., critical for producing sufficient material to crystallize the membrane protein TatC, which is a core component of the twin-arginine protein transport system^22^. This prompted us to characterize Mt56(DE3) in further detail.

In this report, we now describe the isolation and characterization of Mt56(DE3). It is shown that one amino acid substitution in its T7 RNAP is responsible for the improved membrane protein production characteristics of Mt56(DE3).

## Results

### Isolation and initial characterization of the membrane protein production strain Mt56(DE3)

**Figure 1:**
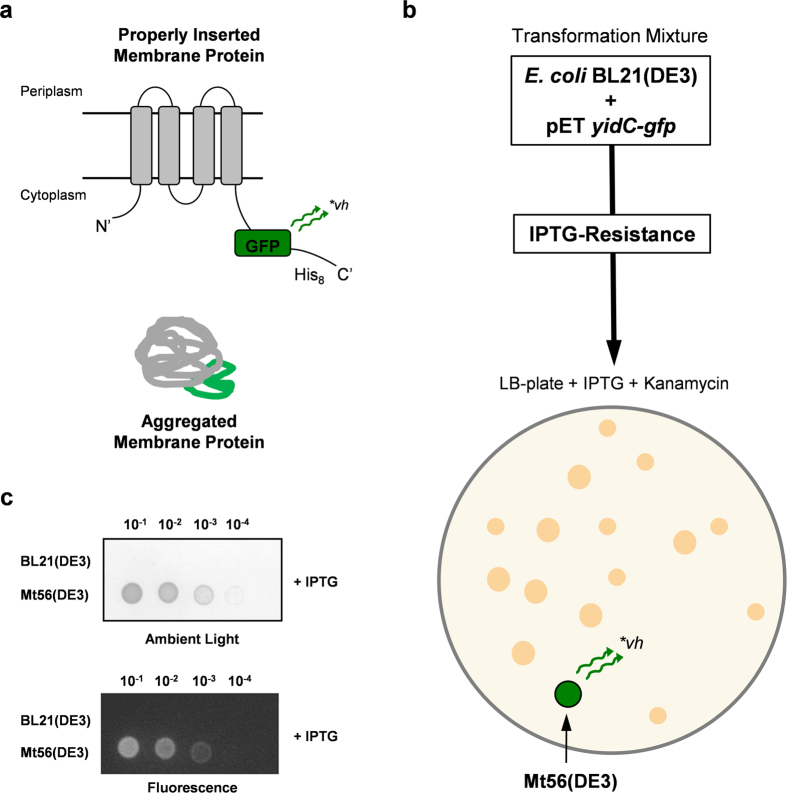
Isolation of Mt56(DE3) from BL21(DE3). The exceptionally toxic target membrane protein YidC, which in contrast to other targets, does upon T7-based production not seem to lead to the isolation of mutations in P_*lac*UV5_ due to recombination with P_*lac*WT_ of the *lac*-operon was used to screen for IPTG-resistant BL21(DE3)-derivatives. YidC was C-terminally fused to GFP to facilitate monitoring its production in the cytoplasmic membrane. (**a**) GFP C-terminally fused to a membrane protein only folds properly and becomes fluorescent if the membrane protein-GFP fusion is inserted in the cytoplasmic membrane^33^. If the membrane protein-GFP fusion aggregates in the cytoplasm, the GFP moiety does not fold properly and does not fluoresce. A membrane protein-GFP fusion with a properly folded GFP moiety runs faster in SDS-PAGE than a membrane protein-GFP fusion with a not properly folded GFP moiety^19^,^26^. (**b**) BL21(DE3) was transformed with a pET-derived *yidC-gfp* expression vector and after recovery of the transformation mixture at 37 °C it was plated on LB-agar plates containing the inducer IPTG (0.4 mM). Plates were incubated at 37 °C. The screen yielded quite a number of IPTG resistant colonies. The use of a T7-based *yidC-gfp* expression vector will not result in IPTG-resistant BL21(DE3)-derivatives with a weakened *lac*UV5 promoter governing the expression of *t7rnap*^17^. The only IPTG resistant colony that was fluorescent was the 56^th^ colony monitored for fluorescence. Therefore, the strain derived from this colony was denoted Mutant56(DE3), hereafter referred to as Mt56(DE3). (**c**) Cured Mt56(DE3) was retransformed with the *yidC-gfp* expression vector used for its isolation and, using its ancestor BL21(DE3) transformed with the *yidC-gfp* expression vector as a control, a plate spot test was used to check for the ability to grow in the presence of IPTG (top panel) and fluorescence (bottom panel). Fluorescence is an indication for YidC-GFP production in the cytoplasmic membrane). The dilutions spotted are indicated on top of the panels.

Previously, we observed to our surprise that the T7-based production of the membrane protein YidC in BL21(DE3) does not seem to lead to the isolation of IPTG-resistant mutants with a weakened promoter governing *t7rnap* expression^18^. We reasoned that we could take advantage of this observation to isolate BL21(DE3)-derived membrane protein production strains with genetic adaptations different than the ones isolated thus far and possibly leading to further improved membrane protein production characteristics. To facilitate the isolation of mutants that efficiently produce membrane proteins in the cytoplasmic membrane GFP was C-terminally fused to YidC. It has been shown that when a membrane protein GFP-fusion is integrated in the cytoplasmic membrane of *E. coli* its GFP moiety folds properly and becomes fluorescent, whereas when a membrane protein GFP-fusion ends up in inclusion bodies its GFP moiety does not fold properly and, consequently, is not fluorescent (Fig. 1a). Thus, in contrast to the screens used to isolate C41(DE3) and C43(DE3) we not only screened for IPTG resistance, but also for integration of the produced target protein in the cytoplasmic membrane.

To isolate BL21(DE3)-derived strains with further improved membrane protein production characteristics, the strain was transformed with a pET-derived *yidC-gfp* expression vector. Subsequently, the transformation mixture was plated on LB-agar plates containing the antibiotic kanamycin to maintain the expression vector and the inducer IPTG. The screen yielded quite a number of IPTG resistant colonies. Next, we monitored if these colonies were also fluorescent. All IPTG-resistant colonies, but one, were not fluorescent (Fig. 1b). Only the 56^th^ IPTG resistant colony tested was fluorescent and denoted Mt56(DE3). Prolonged cultivations in LB medium containing IPTG but not containing any antibiotic were required to cure Mt56(DE3) from the *yidC-gfp* expression vector. The cured Mt56(DE3) strain was retransformed with the *yidC-gfp* expression vector used for its isolation, to verify that it was indeed a mutant that was IPTG resistant and fluorescent when producing YidC-GFP. Mt56(DE3) cells and as a control BL21(DE3) cells harbouring the *yidC-gfp* expression vector were cultured in LB medium in the absence of IPTG. Subsequently, the cultures were serial diluted and aliquots were spotted on LB plates containing IPTG (Fig. 1c). This showed that Mt56(DE3) in contrast to BL21(DE3) harbouring the *yidC-gfp* expression vector could grow on LB plates containing IPTG and was fluorescent, which suggests that in Mt56(DE3) YidC-GFP is indeed produced in the cytoplasmic membrane.

**Figure 2:**
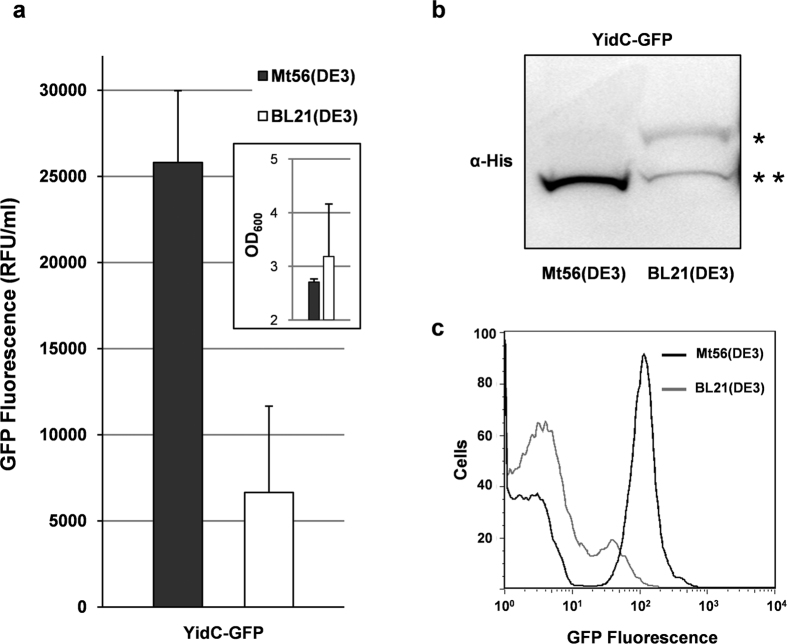
Characterization of Mt56(DE3) producing YidC-GFP. Mt56(DE3) and as a reference its ancestor BL21(DE3) harbouring a pET-derived *yidC-gfp* expression vector were grown in LB medium at 30 °C and *yidC-gfp* expression was induced at an OD_600_ of ~0.4 by the addition of IPTG (0.4 mM). YidC-GFP production and biomass formation were monitored 24 hours after the addition of IPTG. (**a**) YidC-GFP production levels in the cytoplasmic membrane of Mt56(DE3) and BL21(DE3) cells were assessed by monitoring fluorescence (relative fluorescence unit, RFU) per ml of culture. Biomass formation was monitored by measuring the OD_600_ (see the inset). Bars represent the average of three independent experiments. Error bars represent the corresponding standard deviations. (**b**) The ratio of the cytoplasmic membrane inserted to non-inserted YidC-GFP was monitored. Levels of non-inserted (*see also Fig. 1a) and inserted (**see also Fig. 1a) membrane protein-GFP fusions in whole-cell lysates were analyzed by means of SDS-PAGE followed by immuno-blotting using an antibody recognizing the His-tag at the C-terminus of the GFP moiety (see Fig. 1a)^19^,^26^. 0.1 OD_600_ units of cells were loaded per lane. (**c**) The production of YidC-GFP per cell was monitored by flow cytometry. The trace representing Mt56(DE3) cells is black and the trace representing BL21(DE3) cells is in grey.

Also in liquid LB cultures upon the addition of IPTG Mt56(DE3) appeared to produce YidC-GFP to much higher levels than BL21(DE3) (Fig. 2a). To verify if YidC-GFP in Mt56(DE3) was indeed stably produced in the cytoplasmic membrane we used an assay based on SDS-PAGE/immuno-blotting of GFP-fused proteins that allows distinguishing between membrane proteins that are properly inserted in the cytoplasmic membrane and incorrectly folded membrane proteins, which are not inserted in the membrane (Fig. 1a)^19^,^26^. Using this assay we showed that in Mt56(DE3) in contrast to BL21(DE3) YidC-GFP is almost exclusively produced in the cytoplasmic membrane (Fig. 2b). This indicates that the efficient production of YidC-GFP in the cytoplasmic membrane of Mt56(DE3) is hardly affected by the saturation of the membrane protein biogenesis machinery. In contrast, in BL21(DE3) a considerable amount of YidC-GFP was not integrated into the cytoplasmic membrane. It has been shown that saturation of the membrane protein biogenesis machinery is toxic and leads to the accumulation of non-producing cells^17^,^19^. Using flow cytometry it was shown that in a Mt56(DE3)-based culture the vast majority of the cells still produced YidC-GFP, whereas in a BL21(DE3)*-*based culture most cells had stopped producing YidC-GFP (Fig. 2c).

Taken together, in our screen we had isolated a BL21(DE3)-derived strain, Mt56(DE3), that stably produced YidC-GFP in the cytoplasmic membrane to considerably higher levels than BL21(DE3).

### Benchmarking the production of membrane proteins in Mt56(DE3)

**Figure 3:**
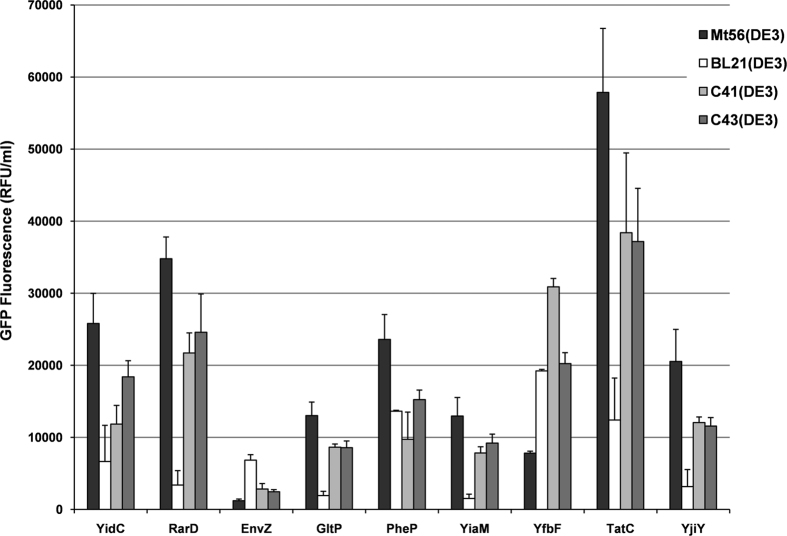
Screening the production of membrane proteins in Mt56(DE3), BL21(DE3), C41(DE3) and C43(DE3). The production of a set of membrane protein GFP-fusions (Supplementary Table 1) was assessed in Mt56(DE3), C41(DE3), C43(DE3) and their ancestor BL21(DE3). Cells were grown in LB medium at 30 °C and target gene expression was induced at an OD_600_ of ~0.4 by the addition of IPTG (0.4 mM). Membrane protein-GFP production was monitored by measuring GFP fluorescence per ml of culture 24 hours after the addition of IPTG (relative fluorescence unit, RFU). Bars represent the average of three independent experiments. Error bars represent the corresponding standard deviations. See for biomass formation Supplementary Fig. 1. For the*E. coli* glutamate proton symporter GltP we verified that the membrane integrated material was also active (results not shown).

To benchmark the production of membrane proteins in Mt56(DE3), we used in addition to YidC-GFP eight more target proteins. To facilitate their detection, also these targets were fused to GFP at their C-termini. The nine targets cover a wide variety of functions, sizes and number of transmembrane segments (Supplementary Table 1). We compared the production yields of all nine targets in the established membrane protein production strains C41(DE3), C43(DE3) to the ones in Mt56(DE3) and their ancestor BL21(DE3). 24 hours after induction of target gene expression, cells were harvested and membrane protein production was monitored (Fig. 3 and Supplementary Fig. 1). Notably, pre-screening membrane protein production yields 4 and 24 hours after IPTG had been added to the cultures indicated that production yields were highest 24 hours after the addition of IPTG. For seven out of the nine targets tested, production yields in Mt56(DE3) were considerably higher than those obtained in C41(DE3), C43(DE3) and BL21(DE3).

Taken together, Mt56(DE3) produced most tested membrane proteins to higher levels than the established membrane protein production strains C41(DE3) and C43(DE3), and their ancestor BL21(DE3). Notably, others have also shown that Mt56(DE3) can produce membrane proteins, including human tetraspanins, to higher levels than C41(DE3) and C43(DE3), and their ancestor BL21(DE3)^22^,^24^. All this prompted us to identify the genetic adaptation(s) responsible for the improved membrane protein production characteristics of Mt56(DE3).

### Sequencing of the Mt56(DE3) genome and phenotypes of its acquired mutations

**Figure 4:**
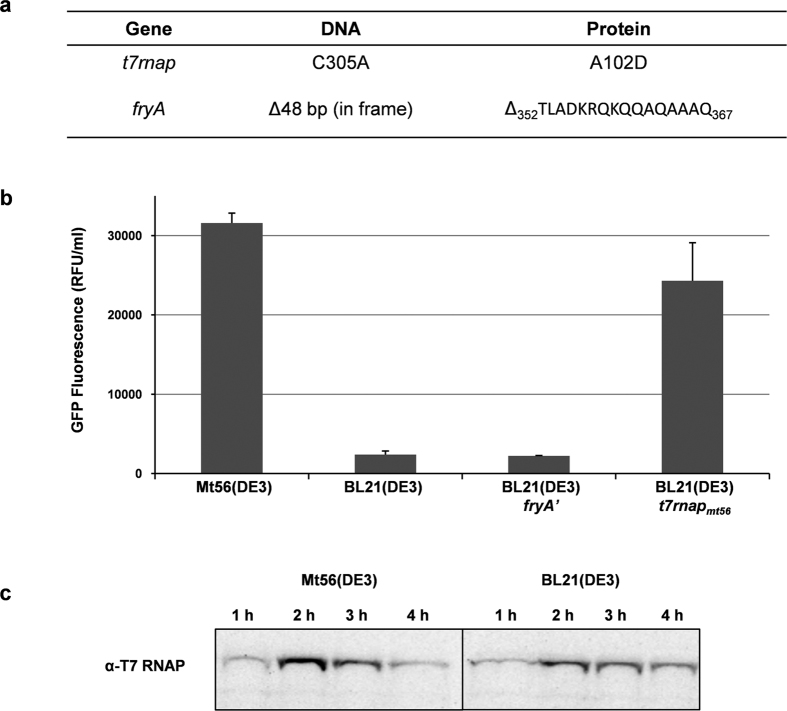
The mutation in *t7rnap* of Mt56(DE3) leads to enhanced membrane protein production. To identify the mutations in Mt56(DE3) its whole genome was sequenced and compared to the sequence of its ancestor BL21(DE3). (**a**) Mt56(DE3) had accumulated two mutations during its isolation. In the gene encoding the T7 RNAP in Mt56(DE3) in position 305 a C:G -> A:T transversion had occurred, leading to a single amino acid exchange in T7 RNAP (A102D). The gene encoding the T7 RNAP in Mt56(DE3) is hereafter referred to as *t7rnap*_*mt56.*_ In the *fryA* gene of Mt56(DE3) a 48 bp in-frame deletion had occurred, resulting in *fryA*’. (**b**) The production of YidC-GFP was assessed in Mt56(DE3), BL21(DE3), BL21(DE3)*t7rnap*_*mt56*_ and BL21(DE3)*fryA*’ (see Methods). Cells were grown in LB medium at 30 °C and target gene expression was induced at an OD_600_ of ~0.4 by the addition of IPTG (0.4 mM). Membrane protein-GFP production was monitored by measuring GFP fluorescence per ml of culture 24 hours after the addition of IPTG (relative fluorescence unit, RFU). Bars represent the average of three independent experiments. Error bars represent the corresponding standard deviations. (**c**) Accumulation levels of T7 RNAP in Mt56(DE3) and BL21(DE3) over time as monitored by immuno-blotting blotting. Cells were cultured in LB medium at 30 °C and Mt56 T7 RNAP and wild-type T7 RNAP accumulation levels were monitored over time upon the addition of IPTG (0.4 mM). For a period of 4 hours after the addition of IPTG samples were taken every hour and subsequently analyzed by means of SDS-PAGE followed by immuno-blotting using an antibody against T7 RNAP.

Sequencing the genome of Mt56(DE3) and subsequently comparing it to the one of its ancestor BL21(DE3), revealed that Mt56(DE3) had acquired two mutations during its isolation (Fig. 4a). In the gene encoding the T7 RNAP in Mt56(DE3) in position 305 a C:G -> A:T transversion had occurred, leading to a single amino acid exchange in T7 RNAP (A102D). Hereafter, the gene encoding T7 RNAP in Mt56(DE3) is referred to as *t7rnap*_*Mt56*_. Furthermore, in the *fryA* gene of Mt56(DE3) we found a 48 bp in-frame deletion, resulting in *fryA*’ (Fig. 4a)^27^.

To characterize the mutations identified in Mt56(DE3), we constructed strains carrying only the single mutations: BL21(DE3)*fryA*’ and BL21(DE3)*t7rnap*_*Mt56*_. Subsequently, we monitored the production of YidC-GFP in these two strains and compared the production yields to the ones obtained in Mt56(DE3) and BL21(DE3) (Fig. 4b). The YidC-GFP production yields in Mt56(DE3) and BL21(DE3)*t7rnap*_*Mt56*_ were high and comparable, and the ones obtained in BL21(DE3) and BL21(DE3)*fryA*’ were low. This indicates that the improved membrane protein production characteristics of Mt56(DE3) are due to the mutation in the *t7rnap* gene. Since key to the improved membrane protein production characteristics of the C41(DE3) and C43(DE3) strains is that they synthesize much less T7 RNAP upon the addition of IPTG than BL21(DE3)^17^, we monitored T7 RNAP accumulation levels in Mt56(DE3) and BL21(DE3) over time by means of immuno-blotting (Fig. 4c). In contrast to C41(DE3) and C43(DE3), the T7 RNAP accumulation levels in Mt56(DE3) were not lower than the ones in BL21(DE3) and followed the same trend as in BL21(DE3). This indicates that the mechanism responsible for the improved membrane protein production characteristics of Mt56(DE3) is different from the mechanism responsible for the improved membrane protein production characteristics of C41(DE3) and C43(DE3).

**Figure 5:**
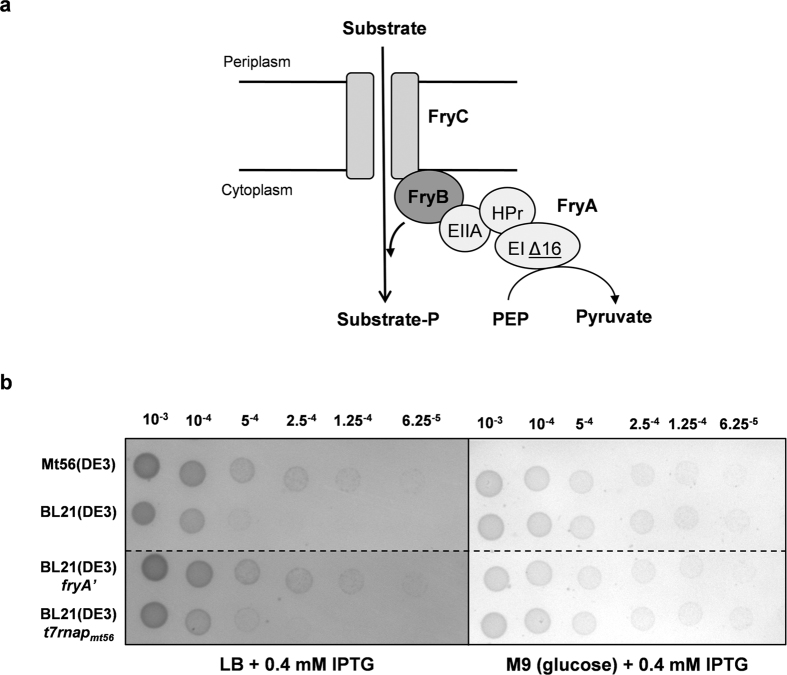
*FryA*’ improves fitness of Mt56(DE3) upon prolonged growth in LB medium. Since *fryA*’ is not involved in the improved membrane protein production characteristics of Mt56(DE3), we explored whether it could have accumulated in response to the repeated prolonged culturing in LB medium during the plasmid curing procedure. (**a**) FryA is a subunit of the putative FryABC transporter. FryA shows homology to the phosphoryl transfer protein PtsA, a subunit of an *E. coli* PEP dependent fructose transporter^27^. In FryA the phosphoryl transfer domains EIA, HPr and EIIA are fused. The 16 amino acid deletion of FryA’ is found in the EIA domain, which catalyses the initial transfer of the phosphoryl group from PEP to the FryABC complex. (**b**) To asses the fitness of Mt56(DE3), BL21(DE3), BL21(DE3)*t7rnap*_*mt56*_ and BL21(DE3)*fryA*’ upon prolonged growth in LB medium containing IPTG, LB/IPTG-cultures were 24 hours after the addition of IPTG serial-diluted in PBS as indicated. Dilutions were subsequently spotted on LB plates containing 0.4 mM IPTG, and as controls dilutions were spotted on LB plates without any IPTG (results not shown; no differences with plates containing IPTG) and M9 minimal medium plates containing 0.2% glucose.

Recently, we have shown that in the BL21(DE3)-derived strain, C41(DE3), several mutations had accumulated during the plasmid curing process^18^. These mutations, which enhance the nutrient uptake capacity of the cell, do not play a role in the improved protein production characteristics of C41(DE3), but rather accumulated during the prolonged growth in the rich medium during the plasmid curing step. So far, no function has been assigned to *fryA*, but the gene is predicted to encode a fused phosphoryl transfer protein as part of the putative PTS permease FryABC (Fig. 5a)^27^. Notably, the 48 bp deletion occurred in frame, leaving an intact open reading frame (Fig. 5a and Supplementary Fig. 2). No residues predicted to be involved in substrate binding or phosphoryl transfer were affected by the deletion (results not shown). We reasoned that the deletion in *fryA* could give a competitive advantage during repeated prolonged cultivations in the rich LB medium. To test this hypothesis we cultured BL21(DE3), Mt56(DE3), BL21(DE3)*t7rnap*_*Mt56*_ and BL21(DE3)*fryA*’ cells for 24 hours in LB-medium. A serial dilution of all four cultures was spotted on LB agar plates (Fig. 5b, left panel). Notably, IPTG was added to mimic the conditions used during the plasmid curing procedure. After 24 hours there were no differences in the OD_600_ values of the cultures. As controls, all serial dilutions were also spotted on minimal M9 medium plates containing glucose as sole carbon and energy source (Fig. 5b, right panel). Strains with the *fryA* deletion grew better on LB plates than the ones without the deletion. On the minimal medium plates with glucose no differences in growth were observed between the four strains, indicating that the amounts of viable cells in all the cultures were similar, which is in keeping with the similar OD_600_ values of all cultures after 24 hours.

In summary, Mt56(DE3) accumulated two genetic adaptations during its isolation from BL21(DE3). The in frame deletion in *fryA* appears to give a competitive advantage during prolonged incubation in LB medium. The single nucleotide substitution in the *t7rnap* gene of Mt56(DE3) leads to a single amino acid exchange in T7 RNAP, which is responsible for the improved membrane protein production characteristics of Mt56(DE3). Interestingly, this mutation does not like the defining mutations in C41(DE3) and C43(DE3) lead to lowered T7 RNAP levels. This indicates that the mechanism responsible for the improved membrane production chracteristics of Mt56(DE3) is different from the one of the C41(DE3) and C43(DE3) strains.

### An A102D amino acid exchange in the T7 RNAP weakens its binding to the T7 promoter

**Figure 6:**
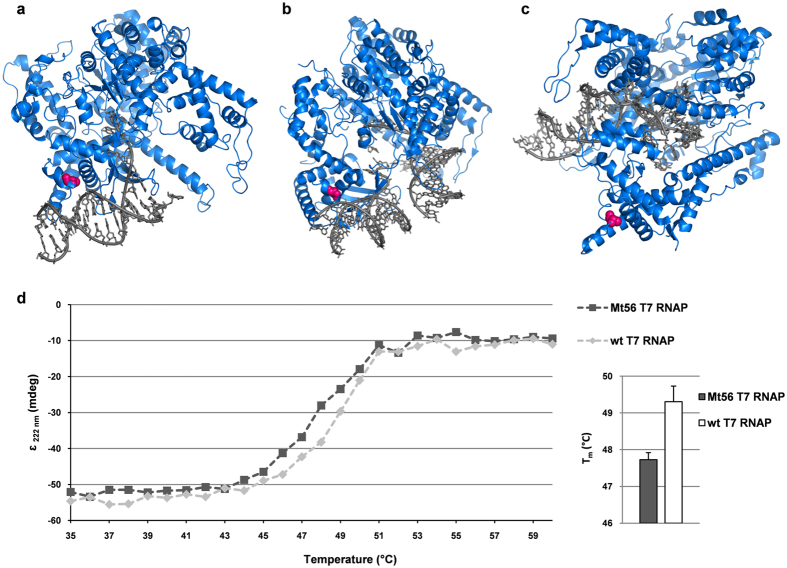
A102D is part of the DNA binding domain of the T7 RNAP and has only a marginal effect on its stability. To assess the potential effect of A102D on the functioning of the T7 RNAP, available structures of the T7 RNAP in complex with DNA were analyzed. The position 102 in the T7 RNAP structure is highlighted using pink spheres. To monitor the effect of A102D on T7 RNAP stability circular dichroism (CD) melting curves were recorded. (**a–c**) From the left to the right; structures representing the T7 RNAP bound to the T7 promoter (PDB 1CEZ), the T7 RNAP in transition from initiation to elongation (PDB 3E2E) and the T7 RNAP elongation complex (PDB 1H38)^28^,^29^,^30^. The structures show that position 102 is part the DNA binding site of the T7 RNAP that is involved in the formation of the initial T7 RNAP-T7 promoter complex, but far away from the active site in the elongation complex. (**d**) Left panel. To monitor the stability of the Mt56 T7 RNAP and wild-type T7 RNAP circular dichroism (CD) melting curves were recorded. CD spectra of the purified wild-type T7 RNAP and Mt56 T7 RNAP were recorded at 222 nm at increasing temperatures. Representative examples are shown. Right panel. Melting temperatures of Mt56 T7 RNAP and wild-type T7 RNAP are based on the CD signal where half the protein was folded. Calculations are based on 3 independent measurements.

The single nucleotide substitution in the *t7rnap* gene of Mt56(DE3) leads to a single amino acid exchange in its T7 RNAP. The mutation in *t7rnap* is responsible for the improved membrane protein production characteristics of Mt56(DE3). However, the mutation does not lead to lowered T7 RNAP levels like the defining mutations in C41(DE3) and C43(DE3) do. Therefore, to further our understanding of the role of the exchange of the alanine in position 102 to an aspartate (A102D) in the T7 RNAP of Mt56(DE3) in improving membrane protein production, we inspected the available crystal structures of different T7 RNAP-DNA complexes. For our analysis we used structures representing the T7 RNAP bound to the T7 promoter (PDB 1CEZ), the T7 RNAP in transition from initiation to elongation (PDB 3E2E) and the T7 RNAP elongation complex (PDB 1H38) (Fig. 6a–c)^28^,^29^,^30^. The structures showed that amino acid residue 102 (highlighted using pink spheres) is positioned within the DNA binding site of the T7 RNAP that is involved in the formation of the initial T7 RNAP-T7 promoter complex (Fig. 6a). This site is also involved in binding of the T7 RNAP to the DNA during the transition of the T7 RNAP-DNA complex from initiation to elongation (Fig. 6b). In the elongation complex position 102 is far away from the active site of the T7 RNAP (Fig. 6c).

Next, we zoomed in on the structure around the alanine in position 102 in the wild-type T7 RNAP. The side chain of this alanine is facing towards the inside of the protein. There, together with the hydrophobic residues W88, L106, and V212 it forms a hydrophobic cluster (Supplementary Fig. 3a). Next, we replaced *in silico* the alanine in position 102 by an aspartate. To do so, the aspartate had to be placed in a conformation such that its side chain faces outwards to minimize unfavorable interactions between its negative charge and the hydrophobic side chains in its vicinity (Supplementary Fig. 3b). Thus, the A102D amino acid exchange may destabilize the Mt56 T7 RNAP. To monitor the structure and stability of the Mt56 T7 RNAP and wild-type T7 RNAP circular dichroism (CD) melting curves were recorded (Fig. 6d). The shapes of the melting curves of the two polymerases were alike. However, the curve of the Mt56 T7 RNAP had shifted slightly towards lower temperatures. Thus, the replacement of the alanine in position 102 with an aspartate in Mt56 T7 RNAP appears to destabilize the protein to some extent, although the effect is very small. This is in keeping with the observation that upon the addition of IPTG the Mt56 T7 RNAP levels in whole cells are not lower than the wild-type T7 RNAP levels in whole cells (Fig. 4c).

**Figure 7:**
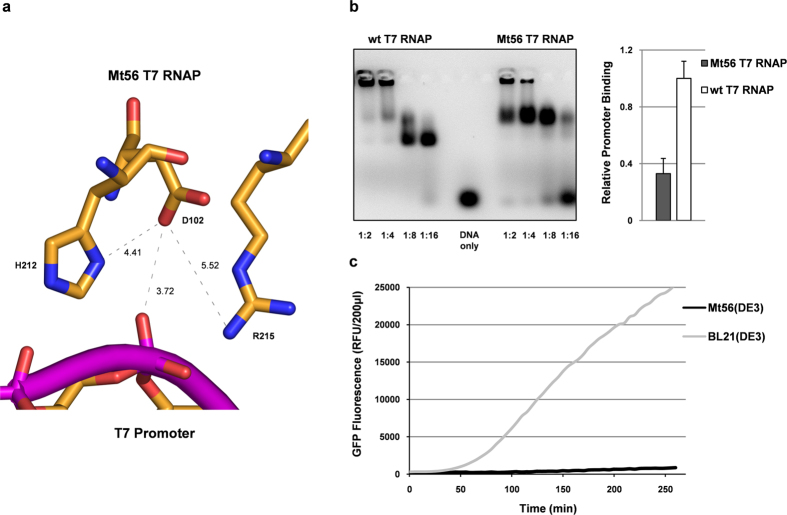
A102D weakens the binding of the T7 RNAP to its promoter and lowers the intensity of target protein production. (**a**) The side chain of the aspartate in position 102 of Mt56 T7 RNAP placed in a conformation that minimizes repelling forces between its negative charge and the hydrophobic side chains in its vicinity (Supplementary Fig. 3). As a consequence a negative charge is positioned in close proximity to the negatively charged phosphate backbone of bound DNA (PDB 3E2E)^28^. The side chain of the aspartate may form salt bridges with the positively charged side chains of H212 and R215 that in the wild-type T7 RNAP facilitate binding to DNA. Positively charged groups are depicted in blue, negatively charged groups in red and the phosphate backbone in purple. Distances are given in Å. (**b**) Using an EMSA the interaction between the wild-type and Mt56 T7 RNAPs, and the T7 promoter was studied. Different amounts of Mt56 T7 RNAP and the wild-type T7 RNAP were mixed with equal amounts of a fluorescently labeled double stranded DNA fragment representing the T7 promoter and subsequently analyzed using agarose gelelectrophoresis (left panel). Primer – protein ratios are indicated. Using broad range titrations the relative binding of the Mt56 T7 RNAP to the T7 promoter was computed (right panel). (**c**) The production of cytoplasmic GFP in Mt56(DE3) and BL21(DE3) was monitored on-line by measuring GFP fluorescence every 5 min in cells cultured in the presence of IPTG in a 96-well plate in a spectrofluorometer.

Based on the structure of the T7 RNAP bound to the T7 promoter it is clear that an aspartate with an outward facing side chain positions a negative charge very close to the phosphate backbone of the DNA (Fig. 7a). This would weaken the binding of the T7 RNAP to the T7 promoter. The aspartate in position 102 may also form a salt bridge with H211 and R215 (Fig. 7a). In the wild-type T7 RNAP these residues facilitate the polymerase - DNA interaction, and the formation of such a salt bridge would further weaken the polymerase - DNA interaction. To test if the aspartate in position 102 of Mt56 T7 RNAP indeed negatively affects the binding of the T7 RNAP to the T7 promoter their interaction was studied using an electrophoretic mobility shift assay (EMSA)^31^,^32^. Different amounts of the wild-type T7 RNAP and Mt56 T7 RNAP were mixed with equal amounts of a double stranded DNA fragment representing the T7 promoter. In the EMSA setup used, the DNA was labelled with a fluorescent dye enabling its detection. The polymerase/DNA mixtures were subsequently analysed using agarose gel electrophoresis. In the mixtures with the wild-type T7 RNAP even at low protein concentrations hardly any free DNA was detected, whereas in the mixtures with the Mt56 T7 RNAP at low protein concentrations a considerable fraction of the DNA was unbound (Fig. 7b). This is an indication that the Mt56 T7 RNAP binds less efficiently to the T7 promoter than the wild-type T7 RNAP. The wild-type T7 RNAP-T7 promoter complex migrates as two bands in the gel. In contrast the Mt56 T7 RNAP-T7 promoter complex migrates only as one band. Next, we monitored the T7 vector-based production of (cytoplasmic) GFP in Mt56(DE3) and BL21(DE3) over time (Fig. 7c). In Mt56(DE3) GFP accumulated over time many times slower than in BL21(DE3), which is in keeping with Mt56 T7 RNAP binding more weakly to the T7 promoter than the wild-type T7 RNAP.

Taken together, the Mt56 T7 RNAP binds less efficiently to the T7 promoter than the wild-type T7 RNAP, thereby lowering the intensity of target protein production.

## Discussion

There are only few reported examples of the isolation of *E. coli* strains with improved membrane protein production characteristics using genetic screens^9^. By far the best known examples are the BL21(DE3)-derived C41(DE3) and C43(DE3) strains^5^. Both these strains have been widely and very successfully used for the production of a variety of membrane proteins^21^. So far, more than twenty years after their isolation no successful attempts have been reported to isolate BL21(DE3)-derivatives with further improved membrane protein production characteristics.

Recently, it was shown that recombination-driven weakening of P_*lac*UV5_ provides BL21(DE3) with a very effective way to alleviate protein production stress in the presence of IPTG^18^. However, when the membrane protein YidC was used no IPTG resistant BL21(DE3)-dervatives with the P_*lac*UV5_ to P_*lac*Weak_ promoter conversion were isolated^18^. Therefore, we reasoned that YidC could be used to isolate IPTG-resistant BL21(DE3)-derivatives that had accumulated mutations enhancing membrane protein production other than the ones weakening P_*lac*UV5_. Importantly, YidC was C-terminally fused to GFP since the GFP-moiety conveniently enabled to screen already on plate for IPTG-resistant BL21(DE3)-derivatives producing YidC-GFP in the cytoplasmic membrane^33^. This enabled us to rapidly filter out IPTG-resistant BL21(DE3) derivatives that somehow had accumulated mutations abolishing YidC-GFP production^34^. Using this approach one mutant, was isolated that was not only IPTG-resistant, but also fluorescent. This mutant was designated Mt56(DE3). Subsequently, its ability to produce YidC-GFP and other membrane proteins was tested, and the production yields were compared with the ones obtained in C41(DE3) and C43(DE3). Most of the yields obtained with Mt56(DE3) were considerably higher than the ones obtained with the C41(DE3) and C43(DE3) strains. As mentioned before, Mt56(DE3) has not only been used by our laboratory to produce membrane proteins, but also others have been using the strain to successfully produce membrane and also soluble proteins for structural and functional studies^22^,^23^,^24^,^25^. Best known examples of membrane proteins produced using Mt56(DE3) are bacterial TatC and human tetraspanins^22^,^24^.

Sequencing the genome of Mt56(DE3) revealed that Mt56(DE3) had accumulated one mutation in the gene encoding the T7 RNAP and another one in *fryA*. In the gene encoding the T7 RNAP in Mt56(DE3) a C:G to A:T transversion, which is a very common mutation in *E. coli*, had led to a single amino acid exchange (A102D)^35^. In the *fryA* gene of Mt56(DE3) there was a 48 bp in frame deletion, resulting in *fryA*’. The deletion in *fryA* could be the result of homologous recombination between the two seven nucleotide long repeats flanking the deleted part of *fryA* or DNA polymerase slippage (Supplementary Fig. 2). Allelic replacement experiments showed that only the mutation in *t7rnap* is responsible for the improved membrane protein production characteristics of Mt56(DE3). Recently, it has been shown that besides the mutations weakening P_*lac*UV5_, C41(DE3) had also accumulated mutations enhancing its nutrient uptake capacity^18^. These mutations do not play any role in the improved membrane protein production characteristics of C41(DE3). Rather, these mutations had accumulated during its prolonged cultivation required for curing the strain from the expression plasmid used for its isolation. It seems likely that during this process cells experienced starvation stress. Also *fryA*’ somehow gives a competitive advantage during the prolonged cultivation step that was used to cure Mt56(DE3) from the expression vector used for its isolation. FryA is predicted to be part of the putative PTS permease FryABC^27^. Due to the similarity of FryA to the EIIA domain of a fructose uptake system, it is very likely that FryA is part of the FryABC transporter. Although the substrate of the putative FryABC transporter is unknown, it is tempting to speculate that the deletion in FryA somehow alters the activity of the transporter, thereby providing Mt56(DE3) with an enhanced capacity of a yet unknown substrate. That might have been advantageous during starvation imposed during the repeated prolonged cultivations to cure Mt56(DE3) from the expression vector used for its isolation.

The point mutation in the gene encoding the T7 RNAP of Mt56(DE3) replaces an alanine by an aspartate at the site of the polymerase that mediates its initial binding with the T7 promoter^28^, and this lowers the affinity of the polymerase for the T7 promoter. The mutation introduces a negative charge close to the promoter-binding site and this likely results in repulsion between the aspartate and the negatively charged phosphate backbone of the DNA. Furthermore, the aspartate is located closely to two positively charged residues (H211 and R215) that are involved in binding of the T7 RNAP to the T7 promoter. The formation of a salt bridge between the aspartate and these residues would further weaken binding of the Mt56 T7 RNAP to the T7 promoter.

The improved membrane protein production characteristics of the C41(DE3) and C43(DE3) strains are due to lowered T7 RNAP levels. However, in Mt56(DE3) accumulation levels of the T7 RNAP are not negatively affected. Thus, the mechanism driving the improved membrane protein production characteristics of Mt56(DE3) is different from the mechanism improving membrane protein production in the C41(DE3) and C43(DE3) strains. Compared to BL21(DE3) both mechanisms decrease membrane protein production intensities. This reduces the saturation of the membrane protein biogenesis pathways and lowers metabolic stress thereby increasing membrane protein production yields in the cytoplasmic membrane^3^,^17^,^19^.

Interestingly, for most membrane protein targets tested, using Mt56(DE3) as a production host resulted in more material produced in the cytoplasmic membrane than when using the C41(DE3) and C43(DE3) strains. These increased levels of recombinant membrane protein in Mt56(DE3) are likely the results of even more favorable production kinetics for the accumulation of the target protein in the cytoplasmic membrane than in the C41(DE3) and C43(DE3) strains.

Previously, we also used an engineering approach to construct a BL21(DE3)-derivative with improved membrane protein production characteristics^17^,^19^. This BL21(DE3)-derivative, Lemo21(DE3), was constructed to mimic the mutations identified in the *lac*UV5 promoter regions governing the expression of *t7rnap* in the C41(DE3) and C43(DE3) strains^17^,^19^. The rationale behind Lemo21(DE3) is that the activity of the T7 RNAP can be modulated by the controlled production of its natural inhibitor, T7 lysozyme. The gene encoding the T7 lysozyme is located on the pLemo plasmid and its expression is under the control of a rhamnose promoter. This promoter is titratable, meaning that the amount of rhamnose added correlates with the amount of protein produced^19^. Thus, the rhamnose promoter covers a broad range of target gene expression intensities. Lemo21(DE3) has been successfully used to optimize the production of a variety of membrane proteins^17^,^19^. Interestingly, Mt56(DE3) also outperforms Lemo21(DE3) for most targets tested (Supplementary Fig. 4). This is another indication that the mechanism that reduces target gene expression can have a significant impact on membrane protein production yields.

Taken together, by promoting the evolution of BL21(DE3) towards enhanced membrane protein production, we isolated Mt56(DE3). For most targets tested Mt56(DE3) outperformed the established membrane protein production strains C41(DE3) and C43(DE3) that were just like Mt56(DE3) isolated in a genetic screen as well as the engineered membrane protein production strain Lemo21(DE3). Our study highlights the potential of using genetics screens for the isolation of strains with further improved membrane protein production characteristics.

## Methods

### Strains, plasmids and culture conditions

*E. coli* strain BL21(DE3) was used for the isolation of Mt56(DE3) (see below)^13^. Mt56(DE3), BL21(DE3), C41(DE3) and C43(DE3) were used for protein production experiments^5^. All genes encoding membrane proteins used in this study as well as the gene encoding GFP were expressed from a pET28a + derived vector as described before^33^. All membrane protein targets were produced as C-terminal GFP-His_8_ fusions as described before^33^. Cells were grown aerobically at 30 °C and 200 rpm in Lysogeny broth (LB) medium (Difco) supplemented with 50 μg/ml kanamycin. Growth was monitored by measuring the OD_600_ with a UV-1601 spectrophotometer (Shimadzu). At an OD_600_ of ~0.4 target gene expression was induced by adding 0.4 mM IPTG. For online GFP fluorescence measurements 200 μl of cultures were transferred after induction with IPTG at an OD_600_ of ~0.4 to a 96 well plate and fluorescence was automatically detected every 5 minutes. The 96 well plate was shaken every 30 seconds^36^.

### Isolation of Mt56(DE3) from BL21(DE3)

To isolate Mt56(DE3) from BL21(DE3), BL21(DE3) was transformed with a *yidC-gfp* expression vector and the transformation mixture was, after recovery at 37 °C for one hour, plated on LB-agar plates containing 50 μg/ml kanamycin and 0.4 mM IPTG^18^. The plates were subsequently incubated at 37 °C^18^. The idea behind the use of the membrane protein YidC C-terminally fused to GFP rather than YidC only was to facilitate the isolation of IPTG-resistant BL21(DE3)-derived mutants efficiently producing YidC in the cytoplasmic membrane^33^. On the transformation plates there was a large number of IPTG resistant BL21(DE3) derivatives. To test if any of these produced YidC-GFP in the cytoplasmic membrane colonies were illuminated with UV light using a standard UV-transilluminator to monitor GFP fluorescence^37^. In our screen all IPTG-resistant colonies, but one, were not fluorescent upon illumination with UV light. The 56^th^ IPTG resistant colony tested was fluorescent and consequently denoted Mt56(DE3). To cure Mt56(DE3) from the *yidC-gfp* expression vector, cells were cultured in LB medium containing IPTG but in the absence of any antibiotic. After 24 hours GFP-fluorescence of the culture was monitored and if the culture was still fluorescent it was backdiluted and cells were cultured for another 24 hours and so on and forth. Seven cycles were required to cure Mt56(DE3) from the *yidC-gfp* expression vector. Subsequently, Mt56(DE3) was retransformed with the *yidC-gfp* expression vector used for its selection and, using BL21(DE3) transformed with the *yidC-gfp* expression vector as a control, a plate spot test was used to check for growth and fluorescence in the presence of IPTG (see below).

### Whole cell fluorescence measurements and flow cytometry

Production of membrane protein GFP fusions was monitored 24 hours after the addition of IPTG using whole-cell fluorescence as described before^36^. Standard deviations are based on a minimum of three biologically independent experiments. GFP fluorescence was analyzed on a single cell level by flow cytometry using a FACSCalibur instrument (BD Biosciences) as described before^36^. FM4-64 membrane staining was used to discriminate between cells and background signal. The FlowJo software (Treestar) was used for raw data analysis.

### SDS-PAGE and immuno-blotting

Whole cell lysates (0.1 OD_600_ units) were analyzed by standard SDS-PAGE using 12% polyacrylamide gels followed by immuno-blotting as described before^36^. His-tagged YidC-GFP was detected using an HRP-conjugated α-His antibody (ThermoFisher) recognizing the C-terminal His-tag. T7 RNAP levels were monitored using a mouse monoclonal antibody (Novagen, recognizing the C-terminal residues 861–883), followed by incubation with a secondary HRP-conjugated goat-α-mouse antibody (Bio-Rad). Proteins were visualized using the ECL-system (GE Healthcare) according to the instructions of the manufacturer and a Fuji LAS-1000 charge coupled device (CCD) camera.

### Sequencing of the genome of Mt56(DE3)

Genomic DNA of Mt56(DE3) was extracted from an overnight culture using QIAamp DNA Mini Kit (QIAGEN, Germany). The genomic libraries were generated using the TruSeq ^®^Nano DNA LT Sample Preparation Kit (Illumina Inc., San Diego CA). Briefly, 100 ng of genomic DNA diluted in 52.5 ul TE buffer was fragmented in Covaris Crimp Cap microtubes on a Covaris E220 ultrasonicator (Woburn, MA) with 5% duty factor, 175 W peak incident power, 200 cycles/burst, and 50 s duration under frequency sweeping mode at 5.5 to 6 °C (Illumina recommendations for a 350 bp average fragment size). The ends of fragmented DNA were repaired by T4 DNA polymerase, Klenow DNA polymerase, and T4 polynucleotide kinase. The Klenow exo minus enzyme was then used to add an ‘A’ base to the 3′ end of the DNA fragments. After the ligation of the adapters to the ends of the DNA fragments, DNA fragments ranging from 300–400 bp were recovered by beads purification. Finally, the adapter-modified DNA fragments were enriched by 3 cycle-PCR. Final concentration of each library was measured by Qubit^®^ 2.0 Florometer and Qubit DNA Broad range assay (Life Technologies). Average dsDNA library sizes was determined using the Agilent DNA 7500 kit on an Agilent 2100 Bioanalyzer. Libraries were normalised and pooled in 10 mM Tris-Cl, pH 8.0, plus 0.05% Tween 20 to the final concentration of 10 nM. Denaturated in 0.2 M NaOH, 10 pm pool of 20 libraries in 600 μl ice-cold HT1 buffer was loaded onto the flow cell provided in the MiSeq Reagent kit v2 300 cycles and sequenced on a MiSeq (Illumina Inc., San Diego CA) platform with a paired-end protocol and read lengths of 151 nt. The raw sequencing data was quality trimmed using the Trimmomatic tool (version 0.32) with the settings CROP:145 HEADCROP:15 SLIDINGWINDOW:4:15 MINLEN:30. From the cleaned data, variants were called using the breseq pipeline (version 0.26.0) with –j 4 (4 CPUs used) and –b 20 (minimum quality score 20) as the only changes to the default settings^38^,^39^. The reference genome for this analysis was *E. coli* BL21(DE3) with the NCBI accession number NC_012971.

### Strain construction

To investigate the role of the two mutations identified in Mt56(DE3) we used a previously described Red-swap-method-based approach to construct strains carrying only a single mutation; *i.e*., BL21(DE3)*t7rnap*_*mt56*_ and BL21(DE3)*fryA*’^18^. In short, the pseudogene *galM* that is in close proximity to the sequence of *t7rnap* in BL21(DE3) and *t7rnap*_*mt56*_ in Mt56(DE3), was used for the integration of a kanamycin cassette into the chromosome of both BL21(DE3) and Mt56(DE3). Notably, deletion of the pseudogene *galM* does not lead to a markedly changed phenotype under the conditions used^40^. Kanamycin-resistant clones were investigated for the proper insertion of the kanamycin cassette by PCR/sequencing. Subsequently, using P1-mediated generalized transduction the wild-type *t7rnap* gene in BL21(DE3) was replaced with *t7rnap*_*mt56*_ of Mt56(DE3) and *t7rnap*_*mt56*_ in Mt56(DE3) was replaced with the wild-type *t7rnap* gene of BL21(DE3)^41^. Upon successful transduction of the genetic region of interest, the kanamycin cassette was removed from the genome of the recipient strain using the FLP-recombinase. The proper deletion of the kanamycin cassette and transfer of *t7rnap* and *t7rnap*_*mt56*_ were verified by PCR/sequencing. The strains that do not contain a kanamycin cassette and are described in this section can be obtained through the SciLifeLab *E. coli* protein production strain collection (https://www.scilifelab.se/platforms/pilot-facilities-projects/). Strains will be made available under an MTA. The costs involved in shipping materials are for the requesting party.

### Spot assays

To characterize the cured Mt56(DE3) strain, it was retransformed with the *yidC-gfp* expression vector used for its selection. An o/n culture of Mt56(DE3) and BL21(DE3) harboring the *yidC-gfp* expression vector (LB supplemented with 0.2% glucose) were serial-diluted in phosphate buffered saline (PBS) as indicated in Fig. 1c. Dilutions were spotted on LB plates containing 50 μg/ml kanamycin and 0.4 mM IPTG, and as a control dilutions were also spotted on LB plates containing kanamycin and 0.2% glucose. GFP-fluorescence was monitored using a UV-transilluminator as described above. To characterize the effect of *fryA*’ on the fitness of cells cultured for a prolonged period of time in LB medium, over night cultures of Mt56(DE3), BL21(DE3), BL21(DE3)*t7rnap*_*mt56*_ and BL21(DE3)*fryA*’ were back diluted 1:50 into LB medium. Cultures were supplemented with 0.4 mM IPTG at an OD_600_ of ~0.4 and grown over night. 24 hours after induction a serial-diluted in PBS as indicated in Fig. 5b. Dilutions were spotted on LB plates containing 0.4 mM IPTG, and as controls dilutions were also spotted on LB plates and M9 minimal medium plates containing 0.2% glucose. A volume of 4 μl was used for spotting. Plates were incubated at 30 °C until colonies became visible.

### In silico analysis of T7 RNAP-DNA complexes

Structures representing the T7 RNAP bound to the T7 promoter (PDB 1CEZ), the T7 RNA polymerase in transition from initiation to elongation (PDB 3E2E) and the T7 RNAP elongation complex (PDB 1H38) from the protein data base were analysed *in silico* using PyMOL (version 0.97)^28^,^29^,^30^. In the structure of the T7 RNA polymerase in transition from initiation to elongation (PDB 3E2E) the alanine in position 102 was replaced by an aspartate using Coot.

### Circular dichroism melting curves

To compare the stability of wild-type T7 RNAP and Mt56 T7 RNAP using circular dichroism (CD) melting curves, His-tagged versions of the proteins were produced using BL21(DE3) from a pET28a + derived plasmid. Four hours after the addition of IPTG cells were harvested and subsequently the His-tagged wild-type T7 RNAP and Mt56 T7 RNAP were isolated using metal affinity chromatography followed by ammonium sulphate precipitation. The precipitated protein was solubilised in an acetate buffer (50 mM Tris acetate, 10 mM magnesium acetate, 50 mM sodium acetate). The concentration of the isolated wild-type T7 RNAP and Mt56 T7 RNAP was determined using the Pierce BCA assay (ThermoFischer). The stability of both wild-type T7 RNAP and Mt56 T7 RNAP was assessed by CD spectroscopy (Chirascan, Applied Photophysics Ltd). The purified protein (0.1 mg/ml) was heated up from 25 °C to 75 °C at a rate of 1 °C per min and the CD signal was recorded at 222 nm (10 measurements per 1 °C). An increase in the CD signal at this wavelength is routinely used to monitor the unfolding of α-helical proteins. The melting temperature was calculated from the CD signal where half the protein is folded.

### Electrophoretic mobility shift assay

An electrophoretic mobility shift assay was used to monitor the interaction between the T7 promoter and the wild-type T7 RNAP and the Mt56 T7 RNAP^31^,^32^. In short, serial dilutions of the isolated proteins were prepared in the aforementioned acetate buffer and they were subsequently incubated in the dark for 30 min at 25 °C with 5′ fluorescently labelled dsDNA (2.5 μM dsDNA, labelled with ATTO488, Eurofins Genomics GmbH) representing the T7 promoter (ATTO488-5′GCCAGTAATACGACTCACTATAGGG3′ and complementary non-labelled primer). Subsequently, standard DNA loading dye (ThermoScientific) was added and the samples were loaded immediately onto a standard 1.5% TAE agarose gel. The gel was run in the dark and upon illumination of the gel using a UV-transilluminator as described above the fluorescent signal of the labelled primer was detected using a CCD camera. Unbound DNA was quantified and plotted against the concentration of wild-type T7 RNAP and Mt56 T7 RNAP. The binding affinity of the wild-type T7 RNAP and the Mt56 T7 RNAP were compared using the half saturated protein concentration. Because high primer concentrations had to be used for detection, this value does not reflect the actual K_d_ of the T7 RNAP but can still be used to compare binding characteristics.

## Additional Information

**How to cite this article:** Baumgarten, T. *et al*. Isolation and characterization of the *E. coli* membrane protein production strain Mutant56(DE3). *Sci. Rep.* **7**, 45089; doi: 10.1038/srep45089 (2017).

**Publisher's note:** Springer Nature remains neutral with regard to jurisdictional claims in published maps and institutional affiliations.

## Supplementary Material

Supplementary Information
