# Supplementary material for: Isolation and characterization of the E. coli membrane protein production strain Mutant56(DE3)
Source: Sci Rep. 2017 Mar 24;7:45089. doi: 10.1038/srep45089 (PMC5364489; doi:10.1038/srep45089)
Supplement: Supplementary Information [file srep45089-s1.pdf]

Supplementary Information

Isolation and characterization of the *E. coli* membrane protein production strain Mutant56(DE3)

Thomas Baumgarten, Susan Schlegel, Samuel Wagner, Mirjam Löw, Jonas Eriksson, Ida Bonde, Markus J. Herrgård, Hermann J. Heipieper, Morten H. H. Nørholm, Dirk Jan Slotboom, Jan-Willem de Gier

Supplementary Table 1. Characteristics of the membrane proteins used for the screen in Fig. 3 and for the screen in Supplementary Figure 4. The information listed; the names of the membrane proteins used, assigned or predicted function, length (number of amino acids) and number of transmembrane helices (TMs). All membrane proteins are from *E. coli* and have a C_{in} topology[1].

Protein	Function	Length (amino acids)	Number of TMs
YidC	inner membrane protein insertase	548	6
RarD	putative multidrug resistance protein	296	9
EnvZ	sensore histidine kinase	450	2
GltP	glutamate/aspartate transporter	437	8
PheP	phenylalanine transporter	458	12
YiaM	2,3-diketo-L-gulonate Na^+ symporter	157	4
YfbF	undecaprenyl phosphate-L-Ara4FN transferase	322	2
TatC	twin arginine protein translocation system	258	6
YjiY	predicted peptide transporter	716	16

Reference

1 Daley, D. O. *et al.* Global topology analysis of the *Escherichia coli* inner membrane proteome. *Science* **308**, 1321-1323 (2005).

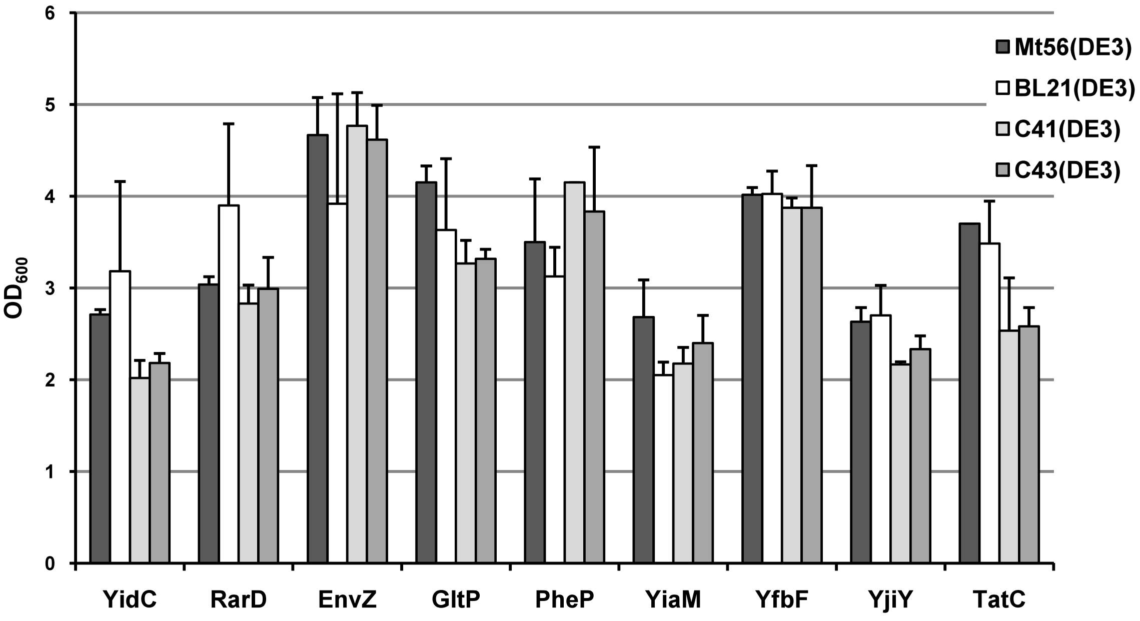

Supplementary Figure 1. Screening the production of membrane proteins in Mt56(DE3), BL21(DE3), C41(DE3) and C43(DE3). Biomass formation in Mt56(DE3), BL21(DE3), C41(DE3) and C43(DE3)-based cultures producing membrane proteins was monitored by measuring the OD_{600} 24 h after the target gene expression was induced. The corresponding target membrane protein production levels can be found in Fig. 3. Bars represent the average of three independent experiments. Error bars represent the corresponding standard deviations.

**BL21(DE3)**	*fryA*	-CAG**CTGCGCG**GCAGCCTGCGCCTGTTGTTTTTGGCGTTTATCCGCCAGCGT**CTGCGCG**ACCTGATAAT-
**Mt56(DE3)**	*fryA'*	-CAG**CTGCGCG**ACCTGATAAT-

Supplementary Figure 2. Sequence comparison of *fryA* from BL21(DE3) and *fryA'* from Mt56(DE3). The gene encoding FryA in Mt56(DE3) has a 48 bp in frame deletion compared to the one in BL21(DE3). The part that is deleted in *fryA'* is underlined in *fryA*. The 7 nt long homologous regions that may have played a role in a recombination event or DNA polymerase slippage leading to the in frame deletion in *fryA'* are in bold.

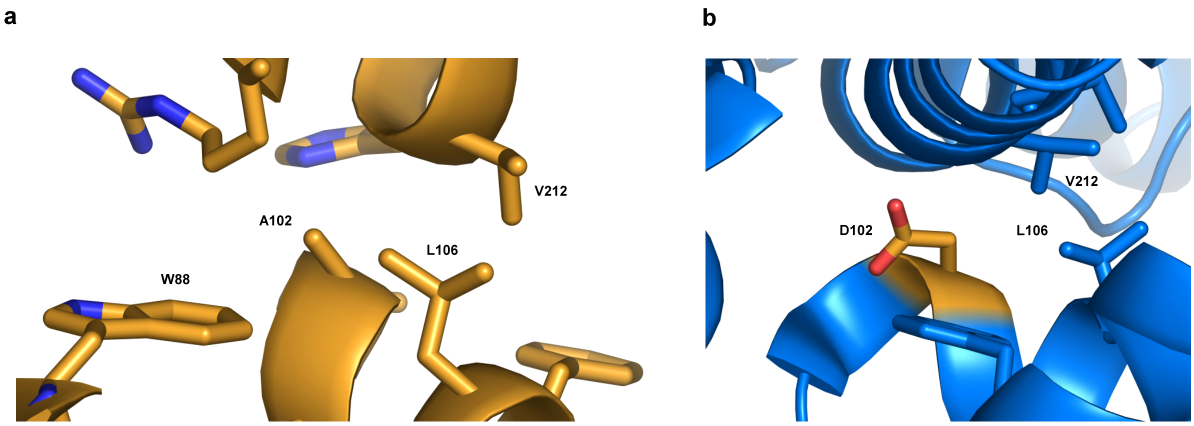

Supplementary Figure 3. Positioning of the side chain of aspartate 102 in the Mt56 T7 RNAP. The alanine 102 in the crystal structure of the wild-type T7 RNAP (PDB 3E2E) was replaced with an aspartate *in silico*. (**a**) Close up of position A102 in the crystal structure of the wild type T7 RNAP. The hydrophobic side chain of A102 forms hydrophobic interactions with L106 and V212, thereby stabilizing helix-helix interactions. (**b**) In the structure of the Mt56 T7 RNAP the aspartate 102 was placed in a position such that unfavorable interactions between its negatively charged side chain and the aforementioned hydrophobic residues are avoided.

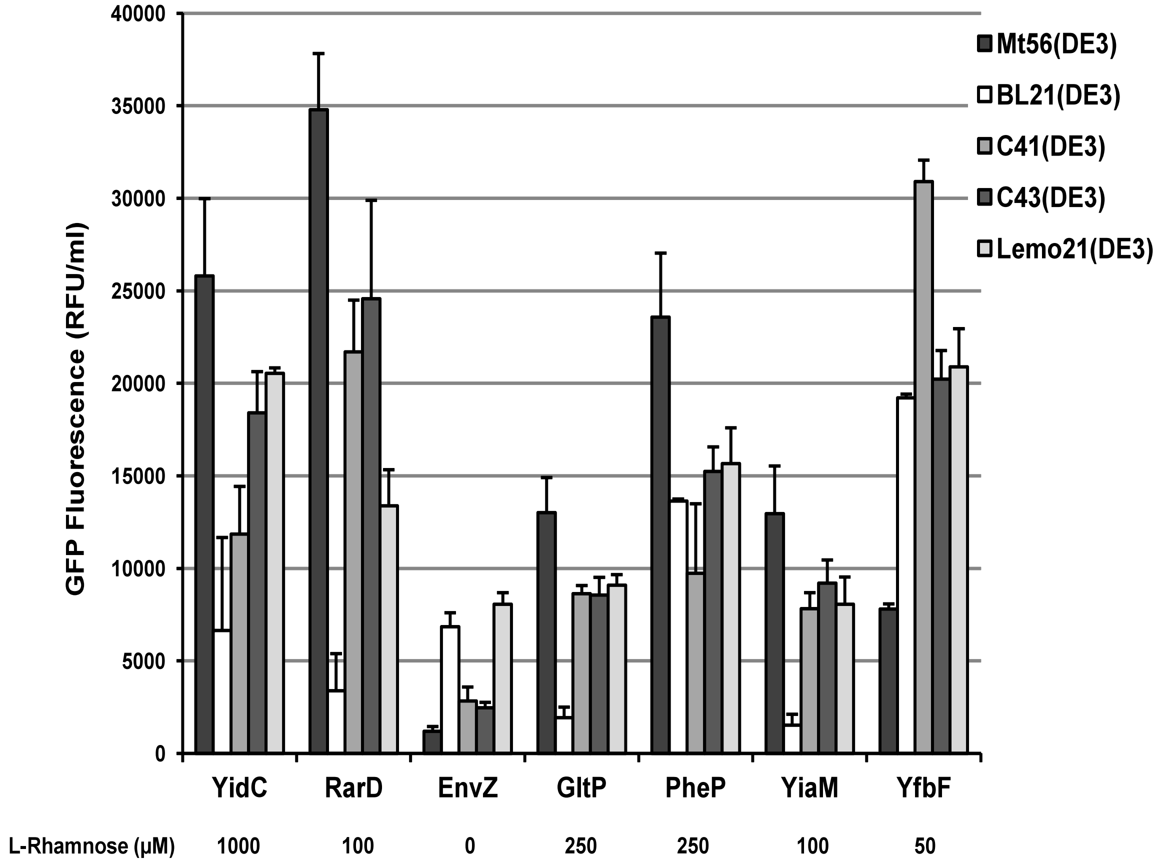

Supplementary Figure 4. Screening the production of membrane proteins in Mt56(DE3), BL21(DE3), C41(DE3), C43(DE3) and Lemo21(DE3). The production of a set of membrane protein GFP-fusions (Supplementary Table 1) was assessed in Mt56(DE3), BL21(DE3), C41(DE3), C43(DE3) and Lemo21(DE3). Cells were grown in LB medium at 30°C and target gene expression was induced at an OD_{600} of ~0.4 by the addition of IPTG (0.4 mM). Membrane protein-GFP production was monitored by measuring GFP fluorescence per ml of culture 24 hours after the addition of IPTG (relative fluorescence unit, RFU). For the production in Lemo21(DE3) the optimal concentration of L-rhamnose is given for each target. Bars represent the average of three independent experiments. Error bars represent the corresponding standard deviations.
